# Physicochemical and Functional Properties of Type I Collagens in Red Stingray (*Dasyatis akajei*) Skin

**DOI:** 10.3390/md17100558

**Published:** 2019-09-28

**Authors:** Junde Chen, Jianying Li, Zhongbao Li, Ruizao Yi, Shenjia Shi, Kunyuan Wu, Yushuang Li, Sijia Wu

**Affiliations:** 1 Technical Innovation Center for Utilization of Marine Biological Resources, Third Institute of Oceanography, Ministry of Natural Resources, Xiamen 361005, China. jyLi9188@163.com (J.L.); yiruizao@163.com (R.Y.); papertiger92@163.com (S.S.); yuzuruhanyu@163.com (K.W.); wwwwaway@163.com (Y.L.); gagasaid@163.com (S.W.); 2 Fisheries College, Jimei University, Xiamen 361021, China; 3 Fujian Provincial Key Laboratory, Marine Fishery Resources and Eco-environment, Jimei University, Xiamen 361021, China

**Keywords:** collagen, fish skin, structure, functional properties, thermal properties, rheology properties

## Abstract

Collagen is widely used in the pharmaceutical, tissue engineering, nutraceutical, and cosmetic industries. In this study, acid-soluble collagen (ASC) and pepsin-soluble collagen (PSC) were extracted from the skin of red stingray, and its physicochemical and functional properties were investigated. The yields of ASC and PSC were 33.95 ± 0.7% and 37.18 ± 0.71% (on a dry weight basis), respectively. ASC and PSC were identified as type I collagen by Sodium Dodecyl Sulfate Polyacrylamide Gel Electrophoresis (SDS-PAGE) analysis, possessing a complete triple helix structure as determined by UV absorption, Fourier transform infrared, circular dichroism, and X-ray diffraction spectroscopy. Contact angle experiments indicated that PSC was more hydrophobic than ASC. Thermal stability tests revealed that the melting temperature of PSC from red stingray skin was higher than that of PSC from duck skin, and the difference in the melting temperature between these two PSCs was 9.24 °C. Additionally, both ASC and PSC were functionally superior to some other proteins from terrestrial sources, such as scallop gonad protein, whey protein, and goose liver protein. These results suggest that PSC from red stingray skin could be used instead of terrestrial animal collagen in drugs, foods, cosmetics, and biological functional materials, and as scaffolds for bone regeneration.

## 1. Introduction

Collagen, the most abundant protein in vertebrates, is a major structural protein in the connective tissue of animal skin and bone, and constitutes about 30% of the total proteins [1]. Twenty-eight types of collagens have been identified, each possessing a unique amino acid sequence, structure, and function [2]. Among the collagen types, type I is the most promising in terms of its marketable prospects [3]. It possesses a stable triple helix structure, which is formed by three α subunits and a right-handed helix. The hydrogen bond between glycine and an amide group in the adjacent α chains is the key to a stable collagen triple helix. In type I collagens, a very short terminal region (telopeptide) is also present, but it does not participate in the triple helix structure [4]. The intra- and intermolecular covalent cross-linking in this region is mainly formed by the oxidative deamination of the ε-NH_2_ groups of lysine and hydroxyllysine residues [5]. The structural characteristics of collagen have allowed researchers to isolate it from raw materials with acetic acid, resulting in acid-soluble collagen (ASC) [6]. Pepsin is also used to digest peptide chains in the telopeptide region of collagen molecules, and the obtained collagen is referred to as pepsin-soluble collagen (PSC) [7]. These two types of collagen have distinct physicochemical properties.

Type I collagen is highly biocompatible and biodegradable and is widely used in the pharmaceutical, tissue engineering, health care product, and cosmetic industries [8]. However, the industrial application of collagen depends heavily on its thermal stability [9]. The rheological properties, water and oil affinity, emulsifying capacity, and foaming capacity of collagen are also important factors that determine the industrial use of type I collagen, and these functional properties are closely related to its structure [10]. Traditionally, collagen is isolated from the skin or bone of pigs, cows, and chickens [11]. Recently, outbreaks of bovine spongiform encephalopathy, foot-and-mouth disease, and avian influenza have made consumers wary of collagen and collagen derivatives from terrestrial animals. Owing to religious reasons, pig collagen is also banned from sale in certain areas [12]. These issues have led to a drastic decline in the market demand of collagen and its derivatives from terrestrial animals. Therefore, a safe alternative collagen source is necessary.

The red stingray (*Dasyatis akajei*) is a fish resource that is abundant in the Northwest Pacific Ocean, mainly along the coastal regions of Japan, South Korea, and China [13]. The skin of the fish is rich in collagen and is an excellent source of raw materials that can be used for the development of collagen products. As such, the use of red stingray skin as an alternative source of collagen may be an effective way to obtain high-value-added products. However, only a few studies have systematically investigated collagen in red stingray skin. Thus, this study aims to isolate and characterize the ASC and PSC found in red stingray skin.

## 2. Results and Discussion

### 2.1. Yield 

ASC and PSC isolated from red stingray skin reached yields of 33.95 ± 0.7% and 37.18 ± 0.71% (on a dry weight basis), respectively. The yields of red stingray skin collagen were higher than samples from Savigny skin collagen (ASC, 3.89%; PSC, 6.74%; on a dry weight basis) [14], loach skin collagen (ASC, 22.42%; PSC, 27.32%; on a dry weight basis) [15], balloon skin collagen (ASC, 4%; PSC, 19.5%; on a dry weight basis) [16], and duck collagen (28.37 ± 0.58, on a dry weight basis) [17]. Additionally, the yield of PSC from red stingray skin was higher than ASC, indicating that the breakdown of covalent cross-links by pepsin digestion of the terminal peptides of collagen results in the dissolution of more collagen triple helix structures by acetic acid, thus providing a higher collagen yield [18].

### 2.2. Structural Characterization

#### 2.2.1. Sodium Dodecyl Sulfate Polyacrylamide Gel Electrophoresis (SDS-PAGE)

**Figure 1:**
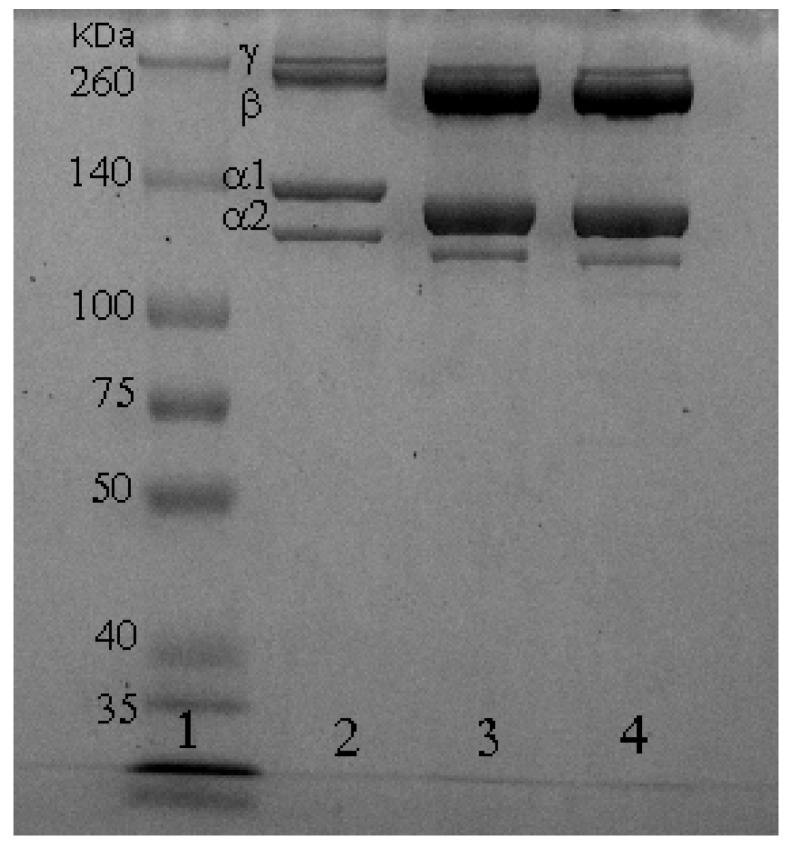
SDS-PAGE patterns of acid-soluble collagen (ASC) and pepsin-soluble collagen (PSC) from the red stingray skin. Lane 1: Standard protein marker (spectra multicolor broad range protein ladder); Lane 2: rat tail type I collagen; Lane 3: ASC collagen; Lane 4: PSC collagen.

The SDS-PAGE protein patterns of red stingray collagens were compared using the profile of commercial rat rail type I collagen, which was used as a reference for the collagen type I protein pattern. Both ASC and PSC contained two different types of α chains (α1 and α2). The gray level ratios of α1/α2 in ASC and PSC were 2.36 and 2.38, respectively, with ratios of α1/α2 close to 2, suggesting that both ASC and PSC belonged to type I collagen [19]. Additionally, these values were similar to type I collagen from the skin of tilapia (α1/α2 = 2.3) [20] and the skin of bighead carp (α1/α2 = 2.13) [19], but higher than type I collagen from rat tail tendon (α1/α2 = 1.84). These results indicated ratios of α1/α2 collagen from different sources might be correlated with the sequence of collagen subunit, temperature of normal habit, and age. Electrophoretic positions of ASC chains (α1-MW, 129 kDa; α2-MW, 119 kDa) were similar to the positions observed for PSC (α1-MW, 129 kDa; α2-MW, 119 kDa), but lower than the positions observed for rat collagen (α1-MW, 137 kDa; α2-MW, 123 kDa). These results indicate that the evolution of collagen genes were potentially the leading cause for the structural differences between terrestrial and aquatic animals [21]. Compared to rat tail tendon collagen, the ASC and PSC from red stingray skin contained more β chains (dimer) and γ chains (trimer), indicating a greater extent of intra- and intermolecular cross-linkage in red stingray ASC and PSC compared to collagen from rat tail tendon (Figure 1) [22].

#### 2.2.2. Secondary Structure

**Figure 2:**
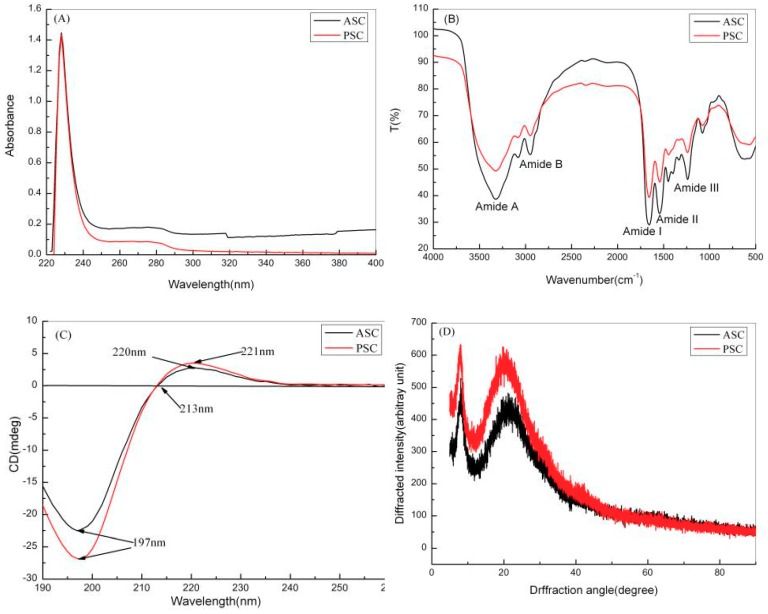
Secondary structure characterization of red stingray collagens. (**A**) UV spectra; (**B**) Fourier transform infrared (FTIR) spectra; (**C**) circular dichroism (CD) spectra; (**D**) X-ray spectra.

UV, Fourier transform infrared (FTIR), circular dichroism (CD), and X-ray diffraction (XRD) spectroscopy were used to identify the secondary structure of red stingray collagens. As shown in Figure 2A, the maximum absorption peaks of both types of collagens appeared at 228 nm. This strong absorption peak was due to electrons absorbing light at 228 nm, as the outer electrons of C=O, COOH, and CO-NH_2_ involved in the peptide bonds of ASC and PSC transit from lower to higher energy states (i.e., n→π*) [9]. ASC and PSC exhibited no absorption peak at 280–300 nm, indicating the absence of tryptophan and tyrosine in the two collagens, as these amino acids exhibit strong absorption in this wavelength range [16].

The characteristic peaks in the FTIR spectra of ASC and PSC correspond to the five amide modes: amide-A, -B, -I, -II, and -III (Figure 2B). The amide-A bands of ASC and PSC appeared at 3320.26 and 3319.96 cm^−1^, respectively, which is due to the N-H stretching vibration of the two collagens and the present hydrogen bonds [23]. According to Doyle et al., the stretching vibration of free N-H appears in the range 3400–3440 cm^−1^, but when the peptide segment of a collagen molecule containing an N-H group participates in hydrogen bonding, the stretching vibration of N-H moves to ~3300 cm^−1^ [23]. The amide-B bands of ASC and PSC appeared at 2936.89 and 2935.55 cm^−1^, respectively, due to the asymmetric stretching vibration of -CH_2_ in ASC and PSC. Muyonga et al. (2004) demonstrated the interactions between collagen amide-I, -II, and -III with collagen C=O stretching, COO^−^ coupling, -NH bending, and C-H stretching [24]. The amide-I, -II, and -III bands of ASC appeared at 1660.19, 1550.68, and 1237.87 cm^−1^, respectively. Compared to ASC, amide-I, -II, and -III bands in PSC appeared at 1659.38, 1551.02, and 1238.79 cm^−1^, respectively, suggesting a higher degree of hydrogen bonding in PSC. The absorbance of the amide-III band of ASC was 0.98, and the band at 1450–1454 cm^−1^ of PSC was 0.96. It was also found in other marine species The absorption ratio between the amide-III and 1450 cm^−1^ peaks was near to 1 (1.01 for salmon and 0.97 for codfish) [25]. With values close to 1, these absorbance values indicate an intact triple helix structure in both collagens [26].

Both species exhibited maximum positive cotton effects at 220 and 221 nm, and maximum negative cotton effects at 197 nm. Zero rotation was observed at 213 nm (Figure 2C). These are characteristics of native collagen, indicating that the ASC prepared in this study contained undissociated collagen helices [27]. According to Feng et al. (1996), the ratio of positive peaks to negative peaks (Rpn) in CD spectra can be used to identify triple helical conformation; the Rpn of collagen with a triple helical structure ranges from 0.12 to 0.15 [28]. The ratio between the absolute values of the positive and negative CD peaks of both ASC and PSC was 0.13, further confirming the intact triple helix structure of the collagens.

Bragg’s Law [d = λ/(2sinθ)], where λ = 0.154 is the X-ray wavelength and θ the Bragg angle, was used to analyze the XRD results. Using the first diffraction peaks of ASC and PSC (peak A1) at 8.05° and 7.94°, respectively, the distances between the molecular chains of ASC and PSC were 1.10 and 1.11 by Bragg’s Law. The distance between PSC molecular chains was greater than that between ASC molecular chains, indicating that pepsin selectively cleaves the terminal peptide sequences of collagen fibers, weakens the interaction between collagen molecules, and increases the distance between collagen molecular chains [29]. Using the second diffraction peaks of ASC and PSC (peak A2) at 21.12° and 19.68°, respectively, the intermolecular distances in ASC and PSC were found to be 0.42 and 0.45 by Bragg’s Law. The intermolecular distance in PSC was greater than in ASC, suggesting that PSC could be potentially more suitable for drug delivery than ASC (Figure 2D) [20].

### 2.3. Physicochemical Properties

#### 2.3.1. Zeta Potential

**Figure 3:**
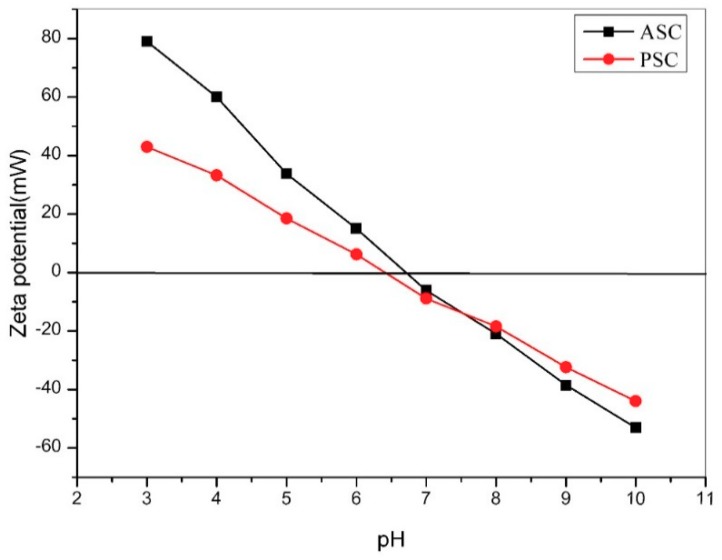
Zeta potential of ASC and PSC at different pH levels.

Both collagens carried positive charges at pH 3–6 and negative charges at pH 7–10. At zero zeta potential, the isoelectric points (pIs) of ASC and PSC were 6.71 and 6.41, respectively. At pH levels above or below the pI of collagen, the repulsion between collagen peptide chains increased, thereby increasing its net solubility (Figure 3). The pI of collagen is related to its amino acid sequence and amino acid residue distribution [20]. The pIs of collagens from different sources were essentially different. The pI values of collagens from red stingray skin were similar to those from brownbanded bamboo shark skin (pI = 6.21, ASC; 6.56, PSC) [30], but higher than those from striped catfish skin (pI = 4.72, ASC; 5.43, PSC) [31] and channel catfish skin (pI = 5.34, ASC; 5.52, PSC) [32].

#### 2.3.2. Water Contact Angle (WCA)

**Figure 4:**
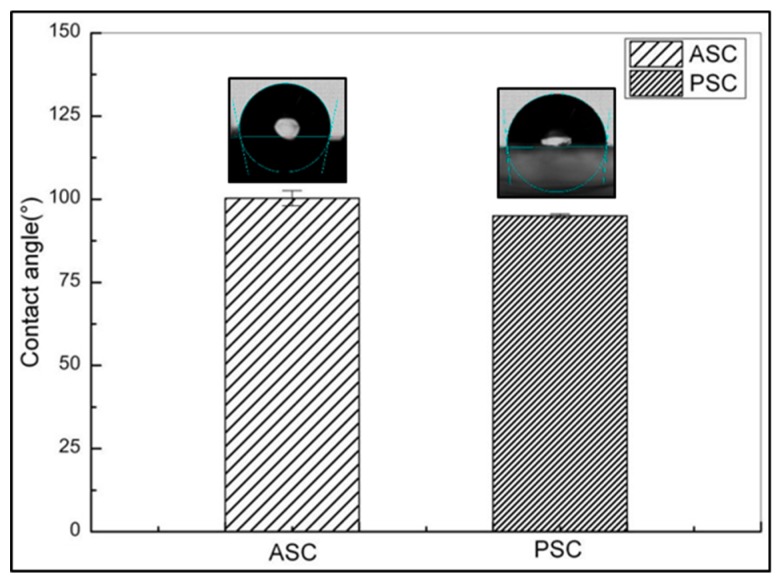
Contact angle data of ASC and PSC from red stingray skin. Values represent means ± standard deviations (SD) of duplicate assays (*n* = 3).

The static WCA of ASC was 100.3° ± 2.31°, and that of PSC was 94.96° ± 0.59°. With values greater than 90°, the contact angles indicate hydrophobicity in both ASC and PSC [33]. The larger static WCA of ASC suggests the presence of a greater number of hydrophobic groups on the surface of ASC than on PSC. According to Zhang et al. [34], the contact angle hysteresis in dynamic contact angle experiments was defined as the difference between advancing and receding angles, which could be used to further characterize the wettability and minimum adhesion at material surface; a larger hysteresis indicates greater hydrophilicity of the material. The contact angle of ASC was 30.67° ± 1.89°, which was smaller than PSC at 42.00° ± 1.14° (Figure 4). The dynamic contact angle experiment reaffirmed the greater hydrophobicity of ASC.

#### 2.3.3. Thermal Properties

**Figure 5:**
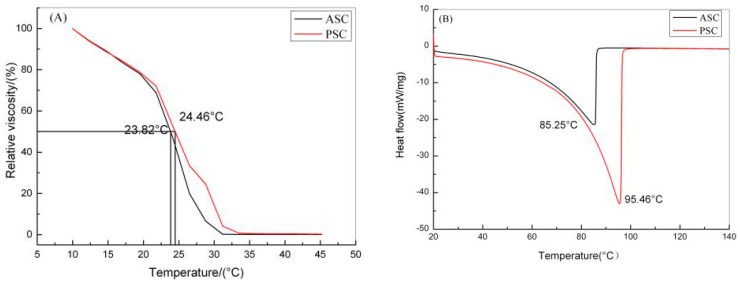
Fractional viscosity (**A**) and differential scanning calorimetry (**B**) of ASC and PSC.

The thermal stability of ASC and PSC were characterized by thermal denaturation temperature (T_d_) and melting temperature (T_m_). T_d_ is the temperature at which the collagen triple helix structure dissociates into random coils in a solution [29]. As reported in Chen et al. [9], the stability of the collagen triple helix is related to the hydrogen bonding in the molecular chains. As the temperature rises, the hydrogen bonds in the collagen molecules are destroyed and the triple helix structure dissociates and turns into random coils. As a result, the viscosity of collagen decreases. As the temperatures increases, the temperature at which the relative viscosity of collagen is 50% is referred to as the thermal denaturation temperature of collagen [35]. The thermal denaturation temperatures of ASC and PSC from red stingray skin were 23.82 °C and 24.46 °C, respectively (Figure 5A). These values were similar to collagens from the swim bladders of miiuy croaker (T_d_ = 24.7 °C, ASC; 26.7 °C, PSC) [35], the skins of tiger puffer (T_d_ = 28.0 °C, ASC; 25.5 °C, PSC) [36], and of Axenillidae (T_d_ = 24.3 °C) [4], but lower than the skins of giant croaker (T_d_ ≈ 34.5 °C, ASC; 34.5 °C, PSC) [37] and of river puffer (T_d_ = 29.5 °C, ASC; 27.5 °C, PSC) [36]. Furthermore, these values were higher than those for the skins of pacific cod (T_d_ = 14.5 °C, ASC; 16.8 °C, PSC) [8] and Spanish mackerel (T_d_ = 15.12 °C, ASC; 14.66 °C, PSC) [38]. T_m_ is defined as the temperature at which the physical form of collagen changes from solid to liquid [39]. At temperatures above T_m_, collagen loses its primary structure. DSC performed on the ASC and PSC from red stingray skin provided melting temperatures of 85.25 °C and 95.46 °C, respectively (Figure 5B). The differences between the T_m_ and T_d_ of ASC and PSC were 61.43 °C and 71 °C, respectively. This could be related to the degree of hydration in the collagens and the number and nature of covalent cross-links [40]. Collagens from terrestrial animals are generally more stable than those from aquatic animals. This stability is also affected by the body temperature of a given organism and the temperature of their habitat [24]. Interestingly, the melting temperature of PSC from red stingray skin was close to that of duck feet (92.48 °C), but higher than both duck skin (86.22 °C) and duck tendon (88.46 °C) [41]. Considering the importance of thermal stability in the application of collagen, we propose that PSC derived from red stingray skin is an excellent possible alternative to terrestrial sources of collagen.

#### 2.3.4. Rheological Characterization of Collagen Solutions

**Figure 6:**
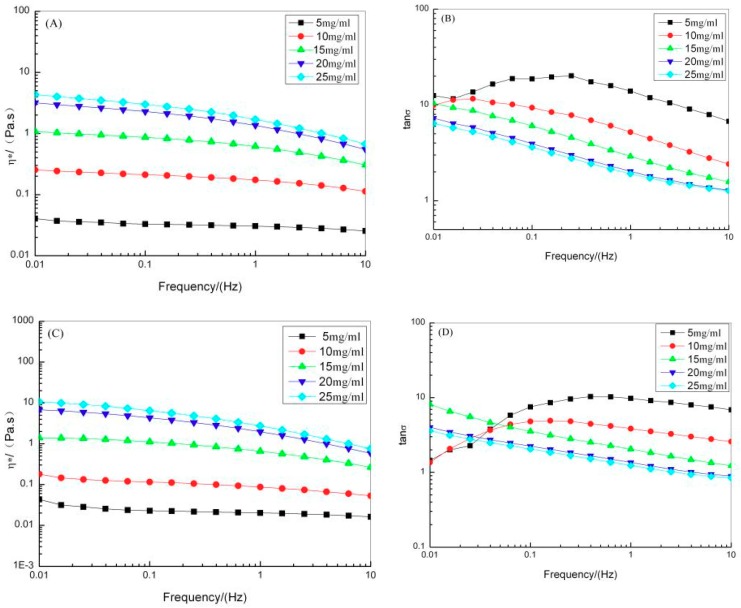
Rheological properties for red stingray collagens solutions with different concentrations. (**A**) complex viscosity (η*) of ASC; (**B**) loss tangents (tanδ) of ASC; (**C**) η* of PSC; (**D**) tanδ of PSC.

As the concentration of ASC and PSC increased from 5 to 25 mg/mL, complex viscosity (η*) increased, indicating that concentration affects the stability of the network structure in ASC and PSC solutions (Figure 6). Additionally, the η* of ASC and PSC decreased as frequency increased, suggesting a shear-thinning flow behavior of the collagen solutions (Figure 6A,C). The loss tangents (tanδ) of ASC and PSC were greater than 1, indicating that the ASC and PSC solutions exhibit viscous behavior (Figure 6B,D). In the frequency range 0.1–10 Hz, tanδ decreased gradually as the ASC and PSC concentration increased. This suggests that as the collagen solution becomes more concentrated, the density of collagen fibers increased and the collagen molecules become more entangled. The motion of collagen molecular chains could not respond rapidly enough to changes in external force; thus, the tanδ of collagen solution gradually decreased [42].

**Figure 7:**
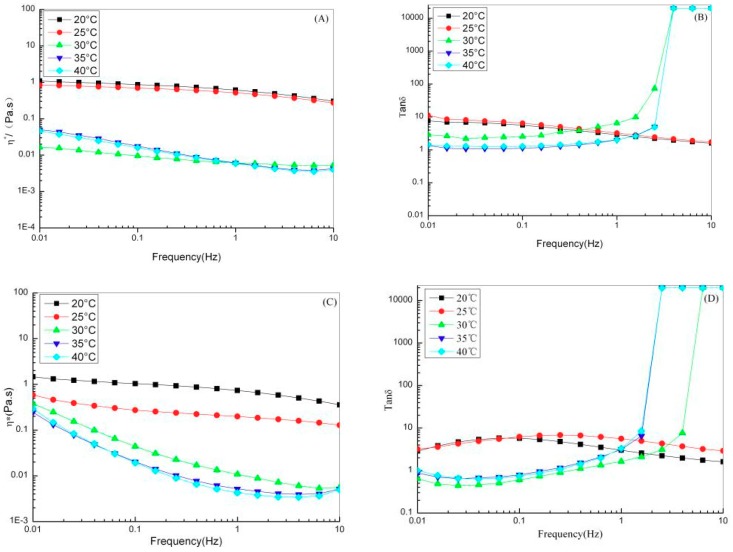
Rheological properties for red stingray collagens solutions at different temperature. (**A**) η* of ASC; (**B**) tanδ of ASC; (**C**) η* of PSC; (**D**) tanδ of PSC.

The η* of the PSC solution decreased gradually when the temperature increased to 20–45 °C and the solution exhibited shear-thinning flow behavior (Figure 7A,C). The η* of the ASC solution exhibited almost no change as the temperature increased to 20–25 °C. As the temperature rose to 30 °C, the η* of the ASC solution decreased drastically. At temperatures above 30 °C, the variation in η* of the ASC solution was no longer regular. The η* values of ASC and PSC exhibited varied rheological behavior as the temperature increased, which is because ASC and PSC have different inter- and intramolecular hydrogen bonds [43]. The tanδ of the ASC solution was greater than 1 at 20–40 °C and the solution exhibited viscous behavior (Figure 7B,D). At temperatures of 20–25 °C, the tanδ of the PSC solution was greater than 1 and the solution exhibited viscous behavior. At temperatures of 30–40 °C and oscillation frequencies of 0.01–0.6 Hz, the tanδ of the PSC solution was less than 1 and the solution exhibited elastic behavior. At temperatures above 30 °C, the change in tanδ of the ASC and PSC solutions lost regularity. This suggests that below its denaturation temperature, collagen could maintain its natural triple helix structure, but above its denaturation temperature, the non-covalent bonds keeping the triple helix structure stable were destroyed. Collagen molecules dissociate, water molecules are released, and the system de-swells. The network structure of the system is thereby destroyed and the molecular structure becomes random and irregular [44].

### 2.4. Functional Properties

#### 2.4.1. Water Absorption Capacity (WAC) and Oil Absorption Capacity (OAC)

The WACs of ASC and PSC were 20.76 ± 0.55 and 28.48 ± 0.69 mL/g, respectively, and were significantly different (*p* <0.05). The collagens from red stingray skin had a higher WAC than scallop gonad protein (6.5 mL/g) [45], glutelin (<11 mL/g) [46], and green tea water-insoluble protein (<3 mL/g) [47]. These differences may arise due to the solubility, particle size, micromorphology of proteins, and the physicochemical environment in which the proteins are found [48]. The OAC of red stingray ASC (41.41 ± 0.47 mL/g) was higher than the OAC of PSC (32.40 ± 0.36 mL/g), which was significantly different (*p* <0.05). As demonstrated by Jitngarmkusol et al., the OAC of a protein is related to its non-polar amino acid residues. The hydrophobic interaction between the non-polar amino acids of protein molecules and the hydrocarbon chains of oil determines the OAC of the protein [49]. The OAC values indicated that red stingray ASC contains more non-polar amino acid residues than PSC. This result is consistent with the results of the contact angle experiments, where ASC had more hydrophobic groups than PSC. Collagens from red stingray skin had a higher OAC than scallop gonad protein (5.2 mL/g) [45], glutelin (<2.3 mL/g) [46], and green tea water-insoluble protein (<5 mL/g) [47]. As reported by Maruyama et al., proteins with higher OAC give better shape retention in food, such as meat or candy products [50]; thus, the collagens from red stingray skin could be used in the meat or candy industries. Additionally, both ASC and PSC exhibited higher OAC than WAC, indicating a larger number of hydrophobic groups in red stingray collagens than hydrophilic groups. This is in agreement with the results of the contact angle experiments.

#### 2.4.2. Foaming and Emulsifying Properties

**Figure 8:**
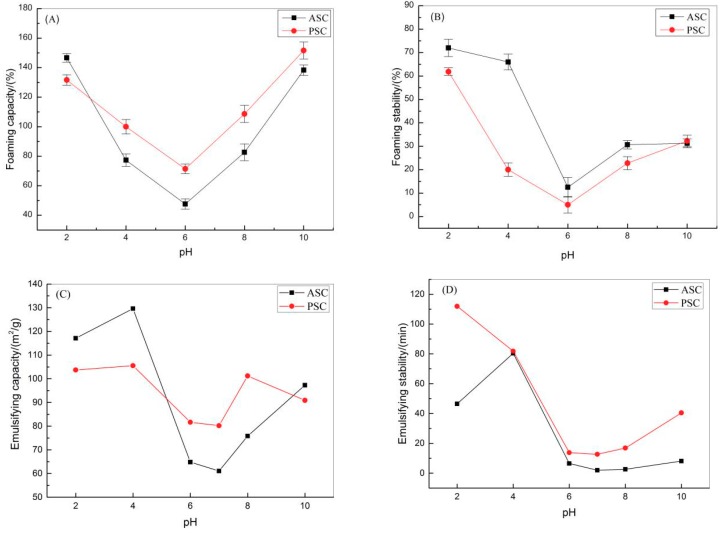
Foaming and emulsifying properties of collagens isolates from red stingray at different pH values. (**A**) foaming capacity (FC); (**B**) foam stability (FS); (**C**) emulsifying activity index (EAI); (**D**) emulsifying stability index (ESI). Values represent means ± standard deviations (SD) of duplicate assays (*n* = 3).

The foaming and emulsifying properties of collagen samples were characterized by foaming capacity (FC), foam stability (FS), emulsifying activity index (EAI), and emulsifying stability index (ESI). The FC of ASC and PSC varied between 47.62 ± 3.50 and 146.67 ± 2.89 and between 71.43 ± 3.20 and 151.67 ± 5.77%, respectively (Figure 8A). The FCs of red stingray skin collagens were higher than those of scallop gonad protein isolates (25–90%) [45], casein (3.95 ± 0.07−10.15 ± 0.21%) [51], and HBC 19 rice bran protein concentrate (5.2 ± 0.28−10.03 ± 0.39%) [51]. The FS of ASC and PSC varied between 12.50 ± 4.17 and 72.00 ± 3.69% and between 5.32 ± 3.55 and 61.85 ± 1.63%, respectively (Figure 8B). The FS values of collagens were higher than those of casein (0.17 ± 0.002–0.54 ± 0.61%), basmati 386 rice bran protein concentrate (0.65 ± 0.02–2.50 ± 0.03%), and HBC 19 rice bran protein concentrate (3.67 ± 0.09–4.30 ± 0.16%) [51]. The FC and FS of collagens decreased at pH 6 near the pI. The low FC and FS could be due to the poor solubility and weak electrostatic repulsion among the collagen molecules, which was insufficiently strong to prevent collagen aggregation molecules. As collagen molecules aggregated, the interaction between protein and water necessary for foaming was weakened and the FC and FS of collagens lowered [46].

The EAI values of ASC and PSC varied between 61.06 ± 0.83 and 117.11 ± 0.25 m^2^/g and between 80.19 ± 0.04 and 105.53 ± 0.41 m^2^/g, respectively (Figure 8C). Meanwhile, the ESI of ASC and PSC varied between 1.96 ± 0.05 and 80.36 ± 0.27 min and between 12.68 ± 0.14 and 111.91 ± 0.57 min, respectively (Figure 8D). The EAI and ESI of ASC and PSC decreased at pH 6–7. At this pH, which is close to the pI, the collagens’ solubility decreased, as did the electrostatic charge on the collagen molecules. The decreased repulsive intensity increased the possibility of oil droplet aggregation. As oil droplets aggregated, the interaction between oil and water necessary for foaming was weakened and the EAI and ESI of collagens lowered [52]. The EAIs of red stingray skin ASC and PSC were higher than jackfruit seed protein isolates (9.09 ± 0.04−9.80 ± 0.04 m^2^/g) [53], whey protein (39.69−65.63 m^2^/g) [54], and goose liver protein (2.3 ± 0.2−3.2 ± 0.1 m^2^/g) [55]. These results suggest that there is potential for red stingray skin to be used as an alternative to terrestrial protein sources. Considering the importance of foaming and emulsifying properties in the food industry, the results of this study suggest that the ASC and PSC from red stingray skin could be applied in baking, beverages, and minor food ingredients.

### 2.5. Cell Proliferation

**Figure 9:**
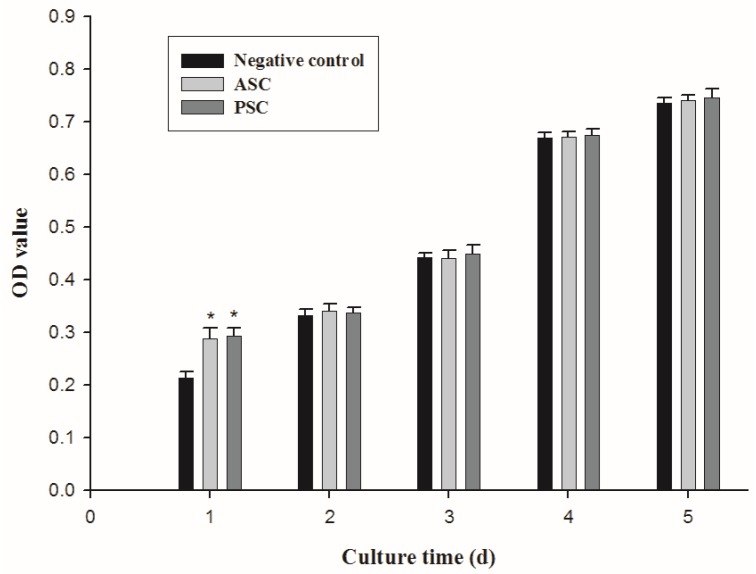
Cell proliferation assay detecting the effects of collagens isolates from red stingray on cell proliferation values represent means ± standard deviations (SD) of duplicate assays (*n* = 3).

Cell proliferation experiments were performed to determine the biocompatibility of NIH3T3 fibroblasts. A higher optical density (OD) value indicated a greater cell proliferation rate [9]. As shown in Figure 9, a significant increase in the OD value in the negative control and collagen samples were found from days 1 to 5 (*p* < 0.05). Additionally, there was a significant difference in OD value between the negative control and collagen samples on day 1 (negative control_1d_, 0.213 ± 0.011; ASC sample_1d_, 0.289 ± 0.020; PSC sample_1d_, 0.293 ± 0.016; *p* < 0.05), suggesting the potential use of ASC and PSC in drugs, foods, cosmetics, and as biomedical materials [56]. However, no significant difference was found between negative control and collagen samples at days 2, 3, 4, and 5. This may be caused by the fact that the denaturation temperatures of ASC and PSC were 23.82 °C and 24.46 °C, while the fibroblasts and collagens were cultured at 37 °C. These phenomena might degrade the collagens’ intact triple helix structure and then lead to loss of collagen activity.

## 3. Materials and Methods

### 3.1. Materials

Red stingray (*Dasyatis akajei*) skin was purchased from an aquatic product processing plant located in Zhangzhou, China. All red stingray experiments were executed according to the protocol approved by Institutional Animal Care and Use Committee of Third Institute of Oceanography, Ministry of Natural Resources. (date of animal research approval, 04 December 2018). Meat and fat layers were removed from the skin by hand. After washing with water, the skin was stored in polyethylene bags and kept at −20 °C for future analyses. *N*,*N*,*N*´,*N*´-tetramethylethylenediamime (TEMED), sodium dodecyl sulphate (SDS), and Coomassie blue R-250 were obtained from Bio-Rad Laboratories (Hercules, CA, USA). High-molecular-weight markers, pepsin, trifluoroacetic acid (TFA), and formic acid were obtained from Sigma Chemical Co. (St. Louis, MO, USA). All other chemicals and reagents used in this study were analytical grade.

### 3.2. Extraction of Collagens

#### 3.2.1. Extraction of ASC

ASC from stingray skin was extracted following the methods previously described by Zhao et al. [35] with modifications. Thawed skin was treated with 10 volumes of 0.1 mol/L NaHCO_3_ for 6 h to remove non-collagenous proteins and pigment. The skin was then stirred and extracted with 0.5 mol/L acetic acid at a 1:10 (*w/v*) ratio for 24 h. Extracted ASC was centrifuged at 20,000× g for 15 min. The supernatant of the ASC extract underwent salt-induced precipitation with 2–5% (*w/v*) NaCl. The precipitate was collected after centrifugation at 10,000× g for 20 min and re-dispersed at a 1:9 (*w/v*) ratio in 0.5 mol/L acetic acid. The solution was dialyzed against a 20-fold volume of 0.1 mol/L acetic acid for 24 h, followed by 24 h of dialysis against distilled water. All processes were carried out below 5 °C. ASC was lyophilized and then stored at −20 °C until use.

#### 3.2.2. Extraction of PSC

PSC from the stingray skin was extracted following the methods previously described by Zhao et al. [35] with modifications. The crushed fish skin was extracted at 4 °C for 24 h in 0.5 M acetic acid containing 1% pepsin (*w/w*) at a 1:10 (*w/v*) ratio. After extraction, the PSC was centrifuged, salt-precipitated, dialyzed, and freeze-dried using the same methods as used for ASC.

#### 3.2.3. Yield

The extraction yields of ASC and PSC were calculated using Equation (1)

(1) Yield/(%)=m1m2×100

where m_1_ is the weight of lyophilized collagen and m_2_ that of initial dry fish skin.

### 3.3. Structural Characterization

#### 3.3.1. SDS-PAGE Analysis

SDS-PAGE was performed following the methods previously described by Laemmli (1970) [57] with modifications using a mini-protein vertical slab electrophoresis system (Bio-Rad Laboratories, Hercules, CA, USA). Samples were dispersed separately in 5% SDS solution. The mixtures were incubated at 85 °C for 1 h and centrifuged at 8500× g for 5 min to remove insoluble substances. The dispersed samples were mixed with sample buffer (0.5 M Tris-HCl, pH 6.8) containing 5% SDS (*w/v*) and 20% glycerol (*v/v*) at a 1:1 (*v/v*) ratio. Samples were loaded onto polyacrylamide gels (8% separating matrix, 3% stacking matrix) and electrophoretically separated under a constant current of 20 mA/gel. The gel was then treated with 25% methanol (*v/v*) and 5% acetic acid (*v/v*) for 30 min and stained for 30 min with 0.1% Coomassie Brilliant Blue R-250 dye (*w/v*) (Bio-Rad Laboratories, Hercules, CA, USA) in 30% methanol (*v/v*) and 10% acetic acid (*v/v*), followed by 30 min of de-staining in 30% (*v/v*) methanol and 10% (*v/v*) acetic acid. The staining ratio of α1 to α2 were analyzed using Quantity One 4.6.0 (Bio-Rad Laboratories, Hercules, CA, USA). High-molecular-weight markers were used to estimate the molecular weight of the samples. 

#### 3.3.2. Spectroscopy Analysis

UV spectroscopy was performed following the methods previously described by Veeruraj et al. [58] with modifications. Samples were dispersed in 0.5 M acetic acid to make 1 mg/mL collagen solutions. The solutions were centrifuged at 18,000 rpm at 4 °C for 5 min to collect the supernatant. The supernatant was placed in a quartz cell to measure the UV-absorption spectra of the ASC and PSC values on a UH5300 UV-vis spectrophotometer (Hitachi Corporation, Osaka, Japan). Using 0.5 M acetic acid solution as the blank, samples were scanned at 400 nm/min with 1-nm data intervals across wavelengths of 220−400 nm.

FTIR spectroscopy was performed following the methods previously described by Chen et al. [20] with modifications. Samples were mixed thoroughly with KBr powder in a moisture-free environment. Samples were subjected to FTIR analysis using a horizontal ATR trough plate crystal cell (PIKE technology Inc., Madison, WI, USA) equipped with a Bruker Model VERTEX 70 FTIR spectrometer (Bruker Co., Ettlingen, Germany). The spectrum was obtained using 32 scans per sample over a range of 500–4000 cm^−1^ at a resolution of 4 cm^−1^. Analyses of the spectral data were carried out using the OPUS v6.5 data-collection software (Bruker Co., Ettlingen, Germany).

CD spectroscopy was performed following the methods previously described by Zhang et al. [59]. Samples were dispersed separately in 0.5 M acetic acid to make 0.1 mg/mL collagen solutions. The solutions were centrifuged at 18,000 rpm at 4 °C for 10 min. The supernatant was collected and placed in a quartz cell to measure the CD spectra of ASC and PSC on a Chirascan CD spectropolarimeter (Applied Photophysics Ltd., Leatherhead, England). The CD spectra were obtained at 15 °C with a time-per-point of 1 s and a wavelength interval of 190–260 nm.

XRD spectroscopy was performed following the methods previously described by Chen et al. [20] with modifications. The crystal structures of lyophilized samples were analyzed using a DX-1000 XRD instrument (Dandong Fangyuan Instrument Co., Ltd., Dandong, China). The X-ray source was Cu *K*α with a tube voltage of 40 kV and tube current of 25 mA, scanning the 2θ interval at 10° and 50° with an angular speed of 0.06°/s.

### 3.4. Physicochemical Properties

#### 3.4.1. Zeta Potential

The zeta potential of the samples was analyzed following the methods previously described by Chen et al. [20] with modifications. Samples were dispersed in 0.5 mol/L acetate solution to a final concentration of 0.05/100 g, and then stirred at 4 °C for 6 h. The zeta potential of ASC samples was measured by a ZetaPALS zeta potential analyzer (Brookhaven Instruments Co., Holtsville, NY, USA). The pH of the samples (20 mL) was adjusted using a BIZTU titration instrument (Brookhaven Instruments Co., Holtsville, NY, USA) across a pH range of 1–11 with 1 mol/L KOH and 1 mol/L nitric acid. pI was determined from a pH exhibiting zero potential.

#### 3.4.2. WCA

Analyses of the static water contact angle were conducted following the methods previously described by Deng et al. [60] with modifications. Samples were fixed on a glass slide and then a drop of ultrapure water was applied to the sample surface. After the droplet was allowed to equilibrate for 3 s, the contact angle of the sample was determined with a DSA 20 contact angle measuring instrument (Krüss Co., Ltd., Hamburg, Germany). Five independent readings were recorded at different locations on the sample to calculate the average contact angle.

Analyses of the dynamic water contact angle were conducted following the methods previously described by Deng et al. [60] with modifications. Samples were fixed on glass slides and 2 μL of ultrapure water were dispensed on the sample surface, forming the initial droplet. The dynamic contact angle of the sample was determined with a DSA 20 contact angle measuring instrument (Krüss Co., Ltd., Hamburg, Germany). The needle of the DSA 20 was placed in the droplet and 6 µL of deionized water were added to the droplet. Changes in the contact angle between the droplet on the sample surface and the sample were video-recorded. Three measurements were recorded on each sample at different positions and the average was calculated.

#### 3.4.3. Thermal Properties

The T_d_ values of the samples were measured following the methods previously described by Zhao et al. [35] with modifications. Samples were dispersed in 0.5 M acetic acid to make 20 mg/mL collagen solutions. The viscosity changes of the collagens at different temperatures (10–45 °C) were measured by an MCR 302 rheometer at a heating rate of 1 °C/min. The temperature at which the relative viscosity was 50% was recorded as the T_d_ of the samples. T_d_ was calculated using Equation (2).

(2) TD=AB×100

where A is the sample viscosity (Pa.s) and B is the sample viscosity (Pa.s) at 10 °C.

T_m_ was assessed following the methods previously described by Huang et al. [61]. Samples were dispersed separately in 0.05 M acetic acid at a 1:40 (*w/v*) ratio and stored at 4 °C for 48 h. Roughly 5–10 mg of swollen collagen samples were added to an empty aluminum pan for differential-scanning-calorimetry (DSC) measurements using the aluminum pan as the blank (the enthalpy of the fusion of indium is 28.451 J/g and the melting point is 156.4 °C). The settings of the differential scanning calorimeter were as follows: temperature range, 20–140 °C; heating rate, 1 °C/min; nitrogen flow in the sample chamber, 50 mL/min. The peak on the DSC spectrum is the T_s_ of collagen.

#### 3.4.4. Rheological Properties

The rheological behavior was analyzed following the methods previously described by Wang et al. [62] with modifications. Oscillatory rheological experiments were conducted using an MCR 302 rheometer (Anton Paar, Gratz, Austria). During all experiments, samples were placed in the rheometer, which was equipped with a cone plate geometry (40 nm diameter, angle 2°) at a gap set of 40 μm. Dynamic frequency sweeps for collagen solutions with different concentrations (5, 10, 15, 20, and 25 mg/mL) were performed from 0.01 to 10 Hz at 25 °C. The effect of temperature on the viscoelastic behavior of the collagens was investigated by inducing a steady-state flow in 15 mg/mL sample solutions with frequencies of 0.01–10 Hz at different temperatures while performing the scan. Each sample was allowed to equilibrate for 10 min at initial temperatures of 0 °C, 25 °C, 30 °C, 35 °C, and 40 °C before measurements were recorded. The functional relationship between η*, tanδ, and oscillation frequency (0.01–10 Hz) was determined.

### 3.5. Functional Properties

#### 3.5.1. WAC and OAC

WAC and OAC were evaluated following the methods previously described by Wani et al. [63] with modifications. Samples were dispersed in ultrapure water/corn oil and mixed thoroughly in a vortex mixer for 2 min. The solution was kept at 25 °C for 30 min and centrifuged at 5,000 rpm/min for 30 min. The supernatant was collected and measured for its volume. WAC and OAC were calculated using Equations (3) and (4).

(3) $$\mathrm{WAC}/(\mathrm{mL}/g)=\left(V_{1}-V_{2}\right)/m$$

where *V*_1_ is the volume of water added during the experiment (mL), *V*_2_ the volume of water left after centrifugation (mL), and m the sample mass (g);

(4) $${\mathrm{OAC}/(\mathrm{mL}/g)=(V}_{1}-V_{2})/m$$

where *V*_1_ is the volume of oil added during the experiment (mL), *V*_2_ the volume of oil left after centrifugation (mL), and m the sample mass (g).

#### 3.5.2. FC and FS

FC and FS were assessed following the methods previously described by Celik et al. [64] with modifications. The sample was dispersed in 0.5 M acetic acid to make a 0.5% (*w/v*) collagen solution. The pH values of 20 mL aliquots of the solution were adjusted to 2, 4, 6, 8, or 10 with 1 M HCl or 1 M NaOH. Each solution was homogenized with a homogenizer at 25 °C at 16,000 rpm/min for 120 s. Then, the sample was quickly transferred to a 250-mL measuring cup. The total volume of the sample was recorded after 30 s. FC and FS were calculated using the following equations:

(5) $${\mathrm{FC}/(\%)=(V}_{1}-V_{2})\times 100/V_{2}$$

(6) $$\mathrm{FS}/(\%)=\left(V_{1}-V_{3}\right)\times 100/(V_{1}-V_{2})$$

where *V*_1_ (mL) is the volume of the sample after homogenization, and *V*_2_ (mL).is the volume before homogenization. *V*_3_ (mL) is the foam volume of the sample after standing at room temperature for 2 h. 

#### 3.5.3. Emulsifying Properties

The EAI and ESI of the samples were determined following the methods previously described by Celik et al. [64]. The sample was dispersed in 0.5 M acetic acid to make a 0.5% (*w/v*) collagen solution. The pH values of 6-mL aliquots of the prepared solution were adjusted to 2, 4, 6, 8, or 10 with 1 M HCl or 1 M NaOH. Corn oil (2 mL) was added to each aliquot and homogenized in a homogenizer for 60 s at 16,000 rpm/min. At 0 and 10 min after homogenization, the emulsion was transferred to a 2-mL centrifugal tube and diluted 100 times with 0.1% (*w/v*) sodium dodecyl sulfate (50-µL sample in 5 mL of 0.1% sodium dodecyl sulfate) with thorough mixing. Absorptions were measured at 500 nm using 0.1% sodium dodecyl sulfate as the blank. EAI and ESI were calculated using the following equations:

(7) $${\mathrm{EAI}/(m}^{2}/g)=(2\times 2.303\times A_{0})/(0.25\times \mathrm{protein}\;\mathrm{weight}(g))$$

(8) $${\mathrm{ESI}(\min)=(A}_{10}\times \Delta t)/(A_{0}-A_{10})$$

where *A*_0_ and *A*_10_ are the absorptions of the emulsions measured at 0 and 10 min, respectively, and *Δt* = 10 min.

### 3.6. Cell Proliferation

Cell proliferation experiments were performed following the methods previously described by Chen et al. [9]. Collagen samples were dispersed in 0.5 M acetic acid to make 0.5 mg/mL collagen solutions. The solutions were added into cell culture plates and then naturally air-dried. The cell culture plates were sterilized via UV treatment and seeded with NIH3T3 fibroblasts. Fibroblasts were incubated with DMEM cell culture medium at 37 °C. Fibroblasts seeded in cell culture plates without collagen were used as negative control, and the cells were grown for 1, 2, 3, 4, and 5 days. At each aforementioned time point, 3-(4,5-dimethylthiazol-2-y)-2,5-diphenyltetrazolium bromide (MTT) solution was added to the cell culture medium and incubated for 4 h at 37 °C. The culture medium was aspirated and then DNSO was added in the plates. After that, the absorbance (OD value) was measured at 490 nm.

### 3.7. Statistical Analyses

Statistical analyses were conducted using SPSS software (SPSS 17.0, Inc., Chicago, 1L, USA). One-way ANOVA analyses followed by Dunnett’s test for multiple comparisons of treatment means with a control were used. Statistical significance was defined as *p* < 0.05.

## 4. Conclusions

In this study, ASC and PSC were isolated from the skin of red stingray and the yield of PSC was found to be higher than ASC. SDS-PAGE analyses confirmed that both collagens belonged to type I collagen. UV, FTIR, CD, and XRD spectroscopy revealed the intact triple helix structure of ASC and PSC, which native collagens possess. The pI of ASC and PSC were 6.71 and 6.41, respectively, as per the zeta-potential analyzer results. Rheological tests showed that both ASC and PSC solutions exhibited shear-thinning flow behavior. In the thermal stability tests, PSC from red stingray skin was found to have a higher melting temperature than duck skin PSC. Contact angle experiments demonstrated the superior hydrophobicity of PSC to ASC. Functional property analyses indicated better functional properties of ASC and PSC than other proteins from terrestrial sources in terms of WAC, OAC, foaming properties, and emulsifying properties. Collectively, these results support the potential use of collagens from red stingray skin as an alternative for terrestrial collagen sources, as well as their potential use in drugs, foods, and cosmetics, and as biological functional materials.
